# Differing calcification processes in cultured vascular smooth muscle cells and osteoblasts

**DOI:** 10.1016/j.yexcr.2019.04.020

**Published:** 2019-07-01

**Authors:** Jessal J. Patel, Lucie E. Bourne, Bethan K. Davies, Timothy R. Arnett, Vicky E. MacRae, Caroline PD. Wheeler-Jones, Isabel R. Orriss

**Affiliations:** a Department of Comparative Biomedical Sciences, Royal Veterinary College, London, UK; b School of Life & Medical Sciences, University of Hertfordshire, Hatfield, UK; c Department of Cell and Developmental Biology, University College London, London, UK; d The Roslin Institute and Royal (Dick) School of Veterinary Studies, University of Edinburgh, Edinburgh, UK

**Keywords:** Vascular calcification, Bone formation, Osteoblast, VSMC, Alkaline phosphatase

## Abstract

Arterial medial calcification (AMC) is the deposition of calcium phosphate mineral, often as hydroxyapatite, in the medial layer of the arteries. AMC shares some similarities to skeletal mineralisation and has been associated with the transdifferentiation of vascular smooth muscle cells (VSMCs) towards an osteoblast-like phenotype. This study used primary mouse VSMCs and calvarial osteoblasts to directly compare the established and widely used *in vitro* models of AMC and bone formation. Significant differences were identified between osteoblasts and calcifying VSMCs. First, osteoblasts formed large mineralised bone nodules that were associated with widespread deposition of an extracellular collagenous matrix. In contrast, VSMCs formed small discrete regions of calcification that were not associated with collagen deposition and did not resemble bone. Second, calcifying VSMCs displayed a progressive reduction in cell viability over time (≤7-fold), with a 50% increase in apoptosis, whereas osteoblast and control VSMCs viability remained unchanged. Third, osteoblasts expressed high levels of alkaline phosphatase (TNAP) activity and TNAP inhibition reduced bone formation by to 90%. TNAP activity in calcifying VSMCs was ∼100-fold lower than that of bone-forming osteoblasts and cultures treated with β-glycerophosphate, a TNAP substrate, did not calcify. Furthermore, TNAP inhibition had no effect on VSMC calcification. Although, VSMC calcification was associated with increased mRNA expression of osteoblast-related genes (e.g. Runx2, osterix, osteocalcin, osteopontin), the relative expression of these genes was up to 40-fold lower in calcifying VSMCs versus bone-forming osteoblasts. In summary, calcifying VSMCs *in vitro* display some limited osteoblast-like characteristics but also differ in several key respects: 1) their inability to form collagen-containing bone; 2) their lack of reliance on TNAP to promote mineral deposition; and, 3) the deleterious effect of calcification on their viability.

## Highlights

• We compare the *in vitro* models of bone formation and vascular smooth muscle cell (VSMC) calcification.
• Osteoblasts form large mineralised bone nodules that are associated with widespread deposition of collagenous matrix.
• Calcifying VSMCs form small discrete regions of calcification not associated with collagen deposition.
• Alkaline phosphatase is essential for bone formation but not VSMC calcification.
• Calcifying VSMCs only display limited osteoblast-like characteristics.

## 1 Introduction

Vascular calcification is a common consequence of ageing, atherosclerosis, diabetes and chronic kidney disease. It is the pathological deposition of calcium phosphate mineral, usually as hydroxyapatite, in the medial and/or intimal layer of the arteries and heart valves. Arterial medial calcification (AMC) refers to the calcification that occurs within the tunica media of blood vessels and is characterised by increased vessel stiffness and reduced blood flow [1]. Traditionally, AMC was thought to be a passive process caused by high serum levels of phosphate and calcium. However, it is now accepted that vascular calcification is a complex cell-mediated process that is thought to share some similarities with physiological bone formation [2]. Indeed, histological analysis of calcified arteries from patients with end stage renal disease has shown the expression of several bone-associated proteins including osteopontin (OPN), tissue non-specific alkaline phosphatase (TNAP) and Runx2 [3,4].

The underlying factors which lead to the development of AMC are multifaceted and not fully characterised. However, there has been significant advances in our understanding in recent years (see reviews [[5], [6], [7], [8]]). The structural protein which predominates in the medial layer of blood vessels is elastin, and it is thought that these fibres could act as the nucleation site for calcification [9]. The predominant cell type driving AMC is the vascular smooth muscle cell (VSMC) [10,11]. Under normal conditions, calcification is prevented by the actions of locally produced (e.g. pyrophosphate) and circulating (e.g. fetuin A) inhibitors [2]. However, when extracellular phosphate levels increase, VSMCs can undergo a phenotypic transdifferentiation to take on characteristics usually associated with bone-forming osteoblasts [8,10,12]. These altered VSMCs have been reported to express TNAP and other bone-related but not bone-specific proteins (e.g. OPN, COL1α1), and release mineralisation-competent matrix vesicles [10,13,14]. Coupled with a reduction in calcification inhibitors and increased VSMC apoptosis, these changes can lead to the development of AMC [2,15].

One of the fundamental regulators of mineralisation is the ratio of pyrophosphate to phosphate within the local environment [16]. Pyrophosphate, which was identified as a key endogenous inhibitor of biomineralisation in the 1960s [17], prevents crystal growth and retards the conversion of amorphous calcium phosphates to crystalline apatites. Pyrophosphate can be generated from nucleotide triphosphates (such as ATP or UTP) by the actions of ecto-nucleotide pyrophosphatase/phosphodiesterases (NPPs). NPPs display widespread tissue expression and NPP1 in particular plays an important role in regulating bone and soft tissue calcification [18,19]. TNAP is the key enzyme involved in pyrophosphate breakdown [20]. Previous work has shown that the opposing actions of NPP1 and TNAP are critical in determining the extracellular phosphate/pyrophosphate ratio and, thus, the level of skeletal mineralisation [20,21]. Increased TNAP expression and activity combined with a decrease in NPP1 expression and activity also play a role in the development of vascular calcification [11,18,[22], [23], [24], [25]].

Despite the reported similarities between AMC and bone formation most *in vitro* studies only examine VSMCs in isolation using increased, and often excessive, phosphate levels as the stimulus for calcification. Furthermore, the classification of a VSMC as an osteoblast-like cell is often based upon the mRNA expression of osteogenic marker genes, many of which are not unique to osteoblasts. This study used the well-established primary mouse VSMC and osteoblast assays to directly compare *in vitro* VSMC calcification and bone formation.

## 2 Methods

### 2.1 Reagents

All tissue culture reagents were purchased from Life Technologies (Paisley, UK); unless mentioned, all chemicals were purchased from Sigma Aldrich (Poole, UK). All primary antibodies were obtained from Abcam UK (Cambridge, UK) and secondary antibodies from Jackson Immuno Research Europe (Ely, UK).

### 2.2 Animals

Primary osteoblasts and VSMCs were isolated from C57BL/6J mice. All procedures complied with the UK animals (Scientific Procedures) Act 1986 and were reviewed and approved by the Royal Veterinary College Research Ethics Committee.

### 2.3 Vascular smooth muscle cell (VSMC) calcification assay

Primary VSMCs were isolated from aortas of 6–8 week old mice. After removal of the adventitia, the aorta was opened to expose the endothelium under a dissection microscope. Tissues from 6 to 8 animals were pooled and incubated with trypsin (0.25% *w/v*) for 10 min to remove any remaining adventitia and endothelium. Tissues were incubated overnight in alpha Minimum Essential Medium, supplemented with 10% foetal calf serum (FCS), 100U/ml penicillin, 100μg/ml streptomycin and 0.25μg/ml amphotericin (complete mixture abbreviated to *α*MEM) before being digested with 425U/ml collagenase type II (Worthington Biomedical Corporation, Lakewood, USA) for 5 h. Isolated VSMCs were expanded in T25 tissue culture flasks in a humidified atmosphere of 5% CO_2_-95% air at 37 °C until confluent. Following seeding into 24-well plates, VSMCs were cultured in either control medium (*α*MEM) or calcifying medium (*α*MEM + 2 mM sodium orthophosphate, 2–10 mM β-glycerophosphate or 1 mM β-glycerophosphate/1 mM sodium diphosphate) for up to 14 days, with half medium changes every 3 days. Medium pH was monitored throughout. All experiments were performed on cells that were expanded and then plated; cells were not passaged at any stage. Key, defined time points for calcifying VSMC cultures were: day 7 (onset of calcification), day 10 (sporadic calcification) and day 14 (widespread calcification).

### 2.4 Osteoblast bone formation assay

Osteoblasts were isolated from the calvariae of 3–5 day old mice by trypsin/collagenase digestion as previously described [[26], [27], [28]]. Following isolation, cells were resuspended in *α*MEM and cultured for 2–4 days in a humidified atmosphere of 5% CO_2_-95% air at 37 °C in 75 cm^2^ flasks until fully confluent. Cells were sub-cultured into 6-well trays in *α*MEM supplemented with 50 μg/ml ascorbic acid and 2 mM β-glycerophosphate or 1 mM β-glycerophosphate/1 mM sodium diphosphate and, with half medium changes every 3 days. Medium pH was monitored throughout. All experiments were performed on cells that were expanded and then plated; cells were not passaged at any stage. Defined time points for osteoblast cultures were day 4 (proliferating cells), day 7 (early osteoblasts), day 10 (mature osteoblasts) and day 14 (mature, bone-forming osteoblasts).

### 2.5 Determination of VSMC calcification and bone formation

Calcifying VSMCs or bone-forming osteoblasts were washed twice with phosphate buffered saline (PBS) and incubated with 0.6 M HCl at room temperature for 24 h. Calcium content was measured colorimetrically by stable interaction with *o*-cresolphthalein using a commercially available kit (Sigma-Adrich, Poole, UK) and corrected for total protein concentration using the Bradford assay (Sigma-Aldrich, Poole, UK). Bone formation in osteoblast cultures was also determined using image analysis, as described previously [26,28]. Calcium deposition was visualised by alizarin red staining of osteoblast/VSMC cell layers, as previously described [28].

### 2.6 Staining for collagen and elastin deposition

Deposited collagen and elastin were visualised in osteoblasts and control/calcifying VSMCs cultured for 7 and 14 days. Cells were fixed for 24 h in Bouin's solution before being stained using an Elastic Stain kit (Sigma Aldrich, Poole, UK) as per manufacturer's instructions. The presence of deposited elastic fibres (black) and/or collagen fibres (red) was visualised by transmitted light microscopy.

### 2.7 Immunofluorescence

Osteoblasts and VSMCs were seeded onto sterile 1 cm diameter discs, cut from Melinex (Dupont Teijin Films, UK) clear polyester film, and cultured for up to 14 days. Upon termination, discs were fixed with 4% paraformaldehyde in 0.1 M phosphate buffer for 20 min at room temperature, washed 3 × 5 min with PBS and stored at 4 °C in PBS until staining. Each disc was incubated with a blocking solution consisting of 2% BSA in PBS, for 1 h. Rabbit primary antibodies were diluted in blocking solution at 1:500 (Col1α1) or 1:200 (SM22α). Discs were incubated overnight in the primary antibody solution at 4 °C. Cells were subjected to three 5 min washes with PBS before incubation for 1 h with the donkey anti-rabbit Cy3-labelled secondary antibody solution (1:400), diluted in PBS with 1% BSA. After three further 5 min PBS washes, discs were mounted on to microscope slides using Prolong™ Diamond Antifade Mountant with DAPI (ThermoFisher Scientific, UK) and viewed by fluorescence microscopy (Cy3 absorbance and emission at 550 nm and 570 nm, respectively).

### 2.8 Elastin and collagen assays

Elastin and soluble collagen levels were investigated in control VSMCs, calcifying VSMCs and osteoblasts after 7 and 14 days of culture. To measure elastin, cell layers were treated with 0.25% trypsin for 10 min to detach the cells. The resulting suspension was spun at 2,500 rpm for 5 min to pellet the cells and associated proteins. Cell-associated elastin was converted to water soluble α-elastin using 0.25 M oxalic acid and heating for 1 h at 100 °C. Elastin levels were measured using the Fastin™ elastin assay (Biocolor Ltd, County Antrim, UK) according to the manufacturer's instructions.

To measure soluble collagen, cells were transferred to αMEM containing 5% FCS and the lysyl oxidase inhibitor β-aminoproponitrile (50 μg/ml) for the final 24 h of culture. The concentration of collagen accumulated in the tissue culture medium was assayed using a Sirius red dye-based kit (Sircol soluble collagen assay, Biocolor Ltd) according to the manufacturer's instructions. For both assays, samples were normalised to total cell protein using the Bradford assay (Sigma-Aldrich, Poole, UK).

### 2.9 Transmission electron microscopy (TEM)

VSMCs and osteoblasts were seeded onto sterile 1 cm diameter discs, cut from melinex clear polyester film (Dupont Teijin Films, UK), and cultured for 14 days in calcifying or non-calcifying conditions. Cells were fixed with fresh 2% glutaraldehyde/0.1 M sodium cacodylate for 48 h. Discs were rinsed with 0.1 M phosphate buffer and post-fixed in 1% osmium tetroxide/1.5% potassium ferrocyanine in 0.1 M sodium cacodylate buffer. Specimens were dehydrated in a graded-ethanol water series and infiltrated with Agar 100 resin. Ultra-thin sections were cut at 70–80 nm using a diamond knife on a Reichert Ultracut E microtome. Sections were collected on 300 mesh grids, counterstained with lead citrate and viewed using a Joel 1010 transition electron microscope; images were recorded using a Gatan Orius CCD camera.

### 2.10 Determination of total TNAP and NPP activity

Enzyme activity was assessed in osteoblasts and VSMCs cultured for 14 days. TNAP activity was measured in cell lysates using a colorimetric assay (Anaspec, CA, USA), as previously described [29]. TNAP activity was normalised to cell protein using the Bradford assay (Sigma-Aldrich, Poole, UK).

The assay to measure total cellular NPP activity was based on the method originally described by Razzell and Khorana [30]. Briefly, cells were lysed in a buffer containing 1% Triton x 100 in 0.2 M Tris base with 1.6 mM MgCl_2_, pH 8.1. Following centrifugation at 500×*g*, the NPP activity of collected supernatants was measured using 5 mM p-nitrophenyl-thymidine 5′-monophosphate as a substrate. Total protein in cell lysates was determined using the Bradford assay (Sigma-Aldrich, Poole, UK).

### 2.11 TNAP staining and inhibition

VSMCs (control and calcifying) and osteoblasts were cultured for 14 days before fixation with 2.5% glutaraldehyde. Cell layers were fixed to demonstrate TNAP expression using a commercially available kit (Sigma-Aldrich, Poole, UK). To determine the effect of TNAP inhibition on calcification and bone formation, calcifying VSMCs and osteoblasts were cultured for 14 days with a 0.1–10 μM TNAP inhibitor (CAS496014-13-2, Merck-Millipore, Watford, UK).

### 2.12 Cell number and viability assay

Cell number and viability were determined at regular intervals during the culture period using the CytoTox 96^®^ colorimetric cytotoxicity assay (Promega UK, Southampton UK), as described previously [29]. Cell supernatants were collected to determine medium lactate dehydrogenase (LDH) levels (cell viability). To establish total cellular LDH levels, cells were lysed with 1% Triton X-100 in water for 1 h. The LDH content of the supernatants and cell lysates were measured colorimetrically (490 nm) according to the manufacturer's instructions. A standard curve for determination of cell numbers was constructed using cells seeded at 10^2^ to 10^6^/well. Cell viability was calculated by expressing medium LDH as a percentage of the total cellular LDH/cell.

### 2.13 Quantification of apoptosis by flow cytometry

VSMCs plated in 24-well trays were cultured in control or calcification medium for 7 days. Apoptosis was assessed via flow cytometry using an annexin V antibody conjugated to fluorescein (Life Technologies, Paisley, UK), as per manufacturer's instructions. Briefly, cells were detached using trypsin (0.25%) and the resultant pellet washed in ice cold PBS. This suspension was centrifuged and resuspended in 1× annexin-binding buffer (Life Technologies, Paisley, UK). A sample of this suspension was incubated with the annexin V antibody for 15 min and then analysed using a BD FACSCanto II Flow Cytometer (Becton, Dickinson and Company, Oxford, UK). Data were processed to calculate percentage apoptosis using Flowing Software (version 2.5.1) (Turku University, Finland).

### 2.14 Total RNA extraction and DNase treatment

VSMCs (control and calcifying) and osteoblasts were cultured for 14 days before total RNA was extracted using TRIZOL^®^ reagent (Invitrogen, Paisley, UK) according to the manufacturer's instructions. Extracted RNA was treated with RNase-free DNase I (35U/ml) for 30 min at 37 °C. The reaction was terminated by heat inactivation at 65 °C for 10 min. Total RNA was quantified spectrophotometrically by measuring absorbance at 260 nM. RNA was stored at −80 °C until amplification by qRT-PCR.

### 2.15 Quantitative real time polymerase chain reaction (qRT-PCR)

VSMC and osteoblast RNA (50 ng) was transcribed and amplified using the qPCRBIO SyGreen one-step qRT-PCR kit (PCR Biosystems, London, UK), which allows cDNA synthesis and PCR amplification to be carried out sequentially. qRT-PCR was performed according to manufacturer's instructions with initial cDNA synthesis (45 °C for 10 min) and reverse transcriptase inactivation (95 °C for 2 min) followed by 40 cycles of denaturation (95 °C for 5 s) and detection (60 °C for 30 s). All reactions were carried out in triplicate using RNAs derived from 4 different cultures. Primers sequences (forward/reverse): ***β-actin****,* S: *gcc ttc ctt cct ggg tat gg*/AS: *tcc gat tca act cat act gc*; ***Collagen type 1 alpha chain 1 (Col1α1):*** S: *ggg aca cag agg ttt cag tgg*/AS: *agc tcc att ttc acc agg act g;* ***TNAP (Alpl)***, S: *aaa cct aga cac aag cac tc*/AS: *tcc gat tca act cat act gc*; ***Osteocalcin (Bglap2)****,* S: *gca gac acc atg agg acc ct*/AS: *gca gct tgt gcc gtc cat ac*; ***Osterix(Sp7)****,* S: *aga gat ctg agc tgg gta gag g*/AS: *aag aga gcc tgg caa gag g*; ***OPN (Spp1)****,* S: *gag agc cag gag agt gcc ga*/AS: *gct ttg gaa ctt gct tga cta tcg*; ***Runx2***, S: *acc ata aca gtc ttc aca aat cct*/AS: *cag gcg atc aga gaa caa act a*; ***Sm22α***, S: *tcc agt cca caa acg acc aag c*/AS: *gaa ttg agc cac ctg ttc cat ctg*; ***Myosin heavy chain (Myh10)***, S: *atc aag cag ctt cgc aaa c*/AS: *tcg agc ctc ttc tag ttc acg*; ***Caldesmon 1 (Cald1)***, S: *cag ctg cgg aca tgc tta g*/AS: *cgt cat cat cat ttc tct gat agg*; ***Smoothelin (Smtn)***, S: *ccg acc aaa cta aca cga aac*/AS: *act cag aat tcc tga cat gtg g*; ***α-Smooth muscle actin (Acta2)***, S: *ctc tct tcc agc cat ctt tca t*/AS: *tat agg tgg ttt cgt gga tgc*; ***Elastin***, S: *tgg agc agg act tgg agg t/*AS: *cct cca gca cca tac tta gca.*

### 2.16 Western blot

Protein was extracted from VSMCs (in control and calcification medium) and osteoblasts at 4, 7 and 14 days. Cell layers were lysed in ice-cold radio immunoprecipitation (RIPA) lysis buffer (50 mM Tris HCl pH 7.4, 150 mM NaCl, 5 mM EDTA, 0.1% SDS 1 mM phenyl methyl sulfonyl fluoride (PMSF), 1 mg/ml aprotinin, 1 mM Na_3_VO_4_ and 2.5 mg/ml deoxicolic acid). Cell homogenates were sonicated for 5 min and stored at −80 °C for at least half an hour before use. Protein concentrations from lysates were determined using the Bradford assay (Sigma-Aldrich, Poole, UK). Prior to loading total protein samples were denatured by incubating at 95 °C for 5 min in the presence of 5× reducing sample buffer (60 mM Tris-HCl pH 6.8, 25% glycerol, 2% SDS, 10% β-mercaptoethanol and 0.1% bromophenol blue). Protein samples (20μg/lane) were loaded into SDS-PAGE (10%) gels and transferred onto a polyvinylidenifluoride (PVDF) membrane (Amersham, Buckinghamshire, UK) by the use of a wet tank blotter (Bio-Rad, Watford, UK) at 150 V for 1 h. Membranes were then blocked with 5% non-fat milk and incubated with β-actin (1:1000), Runx2 (1:500), OPN (1:200), SM22α (1:200) or Acta2 (1:500) antibodies overnight at 4 °C. After washing, blots were incubated in horseradish peroxidase-conjugated secondary antibodies for 1 h at room temperature. A peroxidase detection system (Immobilon™ Western, Merck-Millipore, Watford UK) and ChemiDoc™ XRS + system (Bio-Rad, Watford, UK) was used for the visualisation of immunoreactivity.

### 2.17 Measurement of ATP release

Basal ATP release from VSMCs was measured at 4, 7, 10 and 14 days of culture and from osteoblasts at day 14 of culture. Prior to measurement of ATP release, culture medium was removed, cell layers washed and cells incubated with serum-free DMEM. To measure basal ATP release, samples were collected after 1 h and immediately measured using the luciferin-luciferase assay as described previously [31].

### 2.18 Statistical analysis

Data were analysed using GraphPad Prism 7 software (San Diego, CA). Statistical comparisons were made using a T-test or a one-way analysis of variance (ANOVA) with a post-hoc Bonferroni correction for multiple comparisons. Results are expressed as means ± SEM for 6–12 replicates. Results are representative of experiments performed at least three times using cells obtained from different animal isolations.

## 3 Results

### 3.1 The morphology of *in vitro* VSMC calcification differs from that of bone formation by osteoblasts

**Fig. 1:**
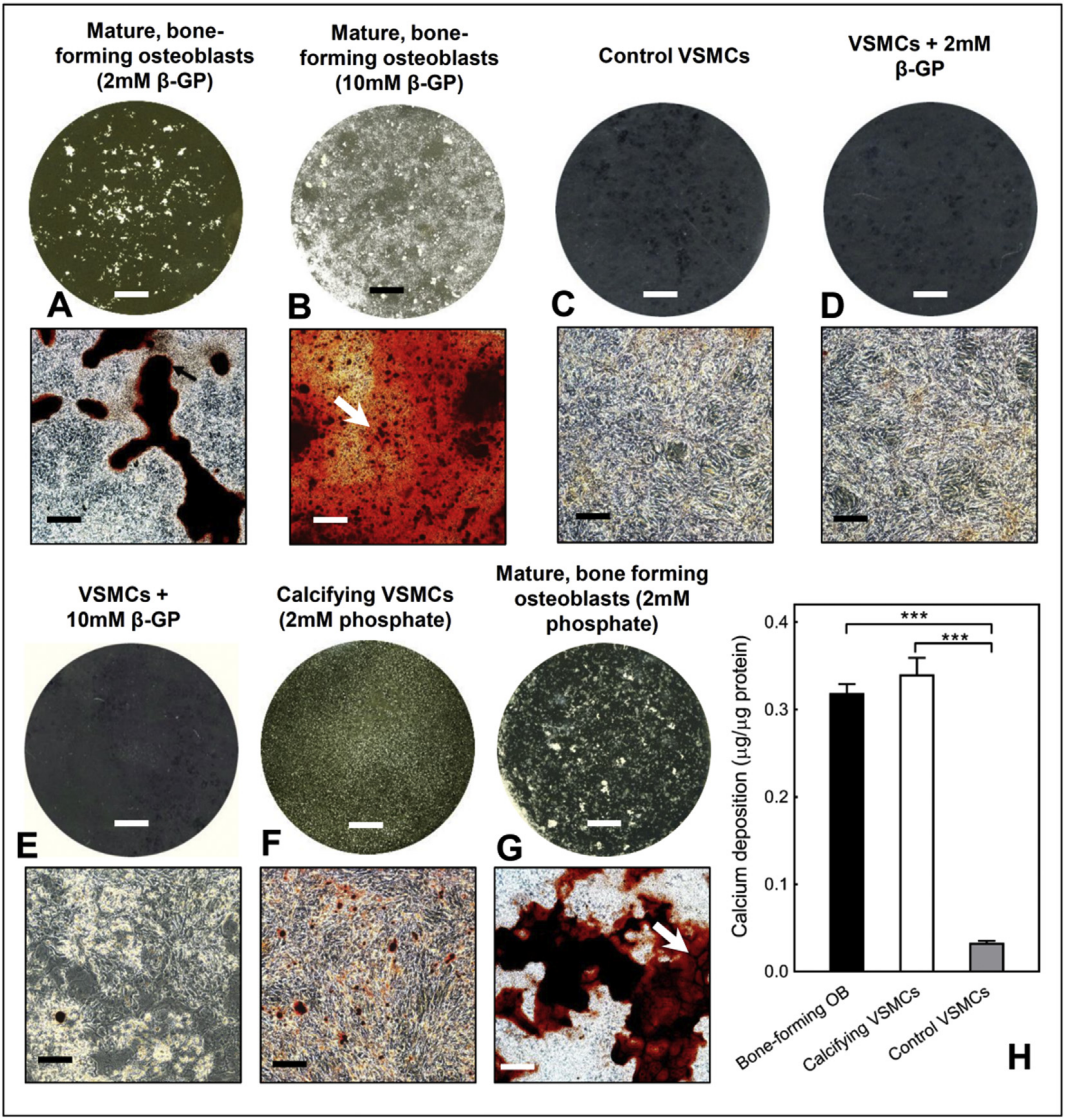
*Comparison of in vitro bone formation and VSMC calcification*. Representative phase contrast microscopy images (alizarin red stained) and whole well scans (unstained) of osteoblast and VSMCs after 14 days in culture. **(A)** Abundant formation of mineralised bone nodules by primary osteoblasts cultured with 2 mM β-glycerophosphate; unmineralised deposited collagen is highlighted by the arrow. **(B)** Widespread, non-specific mineral deposition was observed in osteoblasts cultured with 10 mM β-glycerophosphate (highlighted by the arrow). **(C)** No calcification in control VSMC cultures. VSMCs cultured with **(D)** 2 mM and **(E)** 10 mM β-glycerophosphate for 14 days do not calcify. **(F)** VSMCs grown in 2 mM sodium orthophosphate form small, discrete regions of calcification. These structures are significantly smaller than osteoblast bone nodules and are less visible in the whole well scans. **(G)** Osteoblasts cultured with sodium orthophosphate also display non-specific mineral deposition (white arrow). Scale bars: phase contrast microscopy images = 250 μm, tissue culture wells = 0.5 cm. **(H)** The overall level of deposited calcium was similar in cultures of bone-forming osteoblasts and calcifying VSMCs (2 mM phosphate) but ∼90% lower in control VSMCs. Values are mean ± SEM (*n* = 6 replicate wells), *** = p < 0.001.

Examination of cultures by light microscopy demonstrated that osteoblasts reproducibly formed abundant, large mineralised bone nodules when cultured with 2 mM β-glycerophosphate. These bone structures stain strongly with alizarin red and are often associated with regions of unmineralised collagenous matrix (Fig. 1A). However, excessive levels of β-glycerophosphate (10 mM) resulted in non-specific mineral deposition that is not true bone formation (Fig. 1B). Control VSMCs were densely packed but displayed no signs of calcification (Fig. 1C). VSMCs treated with 2 mM or 10 mM β-glycerophosphate for 14 days did not calcify (Fig. 1D and E). VSMCs cultured with 2 mM sodium orthophosphate formed discrete regions of calcification that were much smaller than osteoblast bone nodules and did not appear to be associated with collagenous matrix (Fig. 1F). In osteoblasts, sodium orthophosphate also resulted in some non-specific mineral deposition (Fig. 1G). Assessment of culture medium pH revealed no significant differences between the different conditions: 2 mM β-glycerophosphate (osteoblasts = pH 7.48 ± 0.01, VSMCs = pH 7.49 ± 0.01), 10 mM β-glycerophosphate (osteoblasts = pH 7.47 ± 0.02, VSMCs = pH 7.46 ± 0.01), sodium orthophosphate (osteoblasts = pH 7.48 ± 0.03, VSMCs = pH 7.48 ± 0.03) and control VSMCs (pH 7.49 ± 0.01).

To ensure that results were not confounded by non-cell mediated calcification processes, unless stated, for all subsequent experiments osteoblasts were cultured in 2 mM β-glycerophosphate and calcifying VSMCs refers to cells cultured with 2 mM sodium orthophosphate. The overall quantity of deposited calcium, as assessed by colorimetric assay, was similar in bone-forming osteoblasts and calcifying VSMCs (Fig. 1H).

### 3.2 Collagen production by osteoblasts and VSMCs

**Fig. 2:**
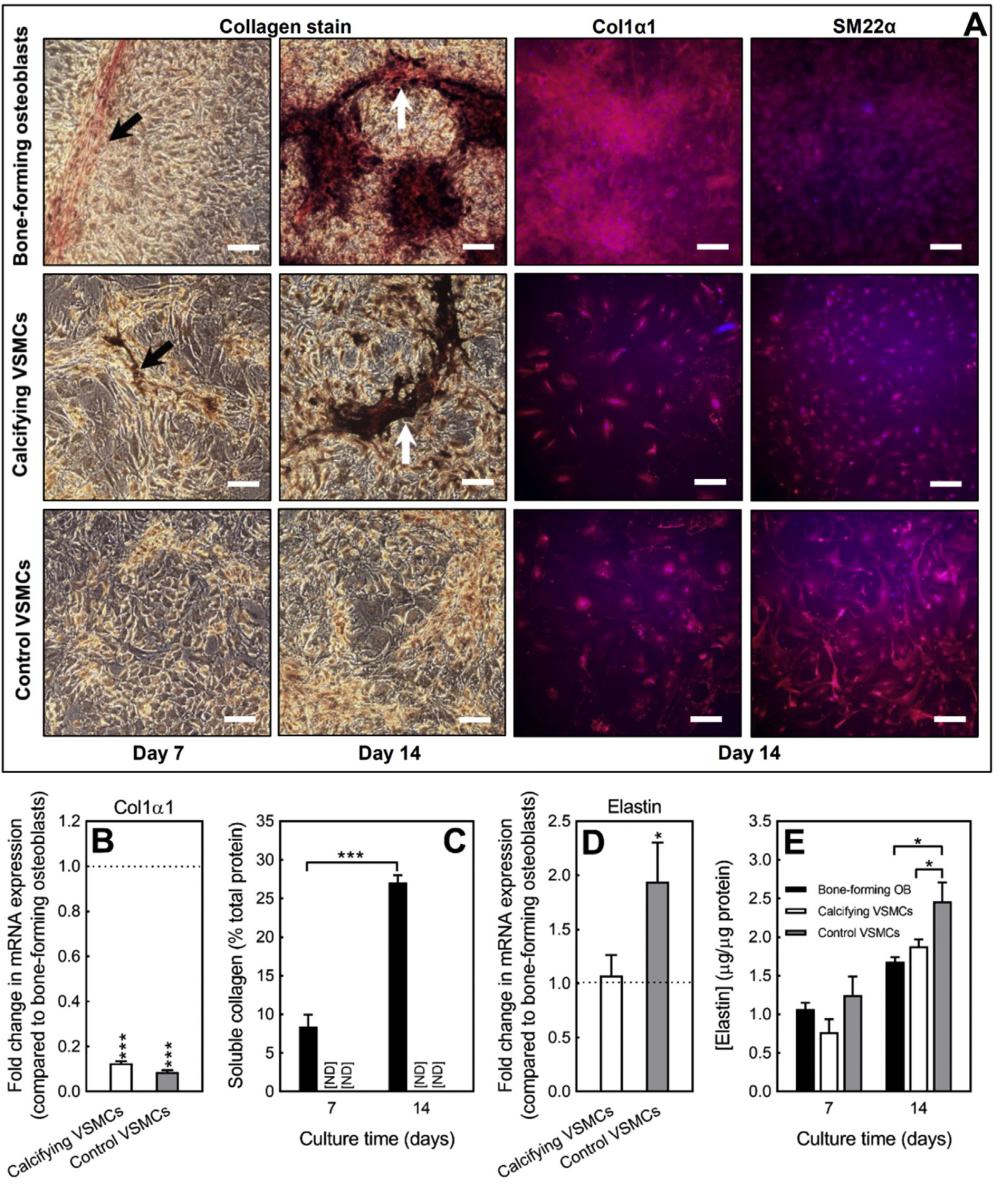
*Collagen and elastin production by osteoblasts and VSMCs*. **(A)** Osteoblasts and VSMCs were cultured for 7 or 14 days and then stained to visualise collagen (red) and elastin (black) fibres. Representative light microscopy images showing widespread deposition of collagen (highlighted by the arrows) in early and mature, bone-forming osteoblasts. Mineralised matrix in bone-forming cultures stained dark brown/black and was associated with collagen staining. No obvious collagen or elastin staining in control VSMCs or calcifying VSMCs via light microscopy. Regions of calcification in VSMC cultures also stained dark brown/black (highlighted by the arrows). Immunofluorescent staining for Col1α1 showed extensive collagen expression and deposition in osteoblast cultures. Control and calcifying VSMCs also expressed Col1α1 protein but immunostaining was primarily cell associated rather than extracellular. Staining for SM22α showed uniform expression across osteoblast and VSMC cell layers. Scale bars, light microscopy = 50 μm, immunofluorescence = 50 μm. **(B)***Col1α1* mRNA expression was up to 11-fold lower in control and calcifying VSMCs, relative to bone-forming osteoblasts. **(C)** Soluble collagen levels increased with osteoblast differentiation and bone formation (black bar). No soluble collagen was detected in control and calcifying VSMC cultures [ND = not detected]. **(D)***Elastin* mRNA expression was ∼2-fold higher in control VSMCs than bone-forming osteoblasts; calcifying VSMCs and osteoblasts displayed similar levels of elastin expression. **(E)** Cell-associated elastin was up to 50% higher in control VSMCs compare to calcifying VSMCs and osteoblasts. Values are mean ± SEM (*n* = 6 replicate wells, *n* = 4 RNA sets), * = p < 0.05, *** = p < 0.001.

Deposition of collagen fibres (red) was visible in both early osteoblast (day 7) and mature, bone-forming osteoblast (day 14) cultures. By day 14, extensive mineralisation of the organic matrix had occurred; collagen fibres showed as regions of red staining underneath the deposited bone mineral (dark brown/black) (Fig. 2A). There was no obvious deposition of collagen fibres in control or calcifying VSMCs at any stage via light microscopy (Fig. 2A). Immunofluoresecent staining for Col1α1 showed extensive protein expression in and around osteoblasts (Fig. 2A). Control and calcifying VSMCs also displayed Col1α1 expression; however, immunostaining was usually cell associated and much less widespread than in osteoblasts (Fig. 2A). Control staining for SM22α showed uniform expression across the cell layers in both VSMC and osteoblast cultures (Fig. 2A).

Analysis of *Col1α1* mRNA levels (day 14 of culture) showed that gene expression was up to 11-fold lower in control and calcifying VSMCs, relative to bone forming osteoblasts (Fig. 2B). Osteoblast differentiation and bone formation were associated with increased levels of soluble collagen. In contrast, no soluble collagen was detected in cultures of control or calcifying VSMCs (Fig. 2C).

### 3.3 Production of elastin by cultured VSMCs and osteoblasts

There was no obvious discrete staining of elastin fibres (black) evident in osteoblast, control or calcifying VSMCs at any stage (Fig. 2A). However, large regions of dark brown/black staining, which corresponded to the areas of calcification, were observed in osteoblasts and calcifying VSMCs. Analysis of *elastin* mRNA levels (day 14 of culture) showed that gene expression was ∼2-fold higher in control VSMCs, relative to bone-forming osteoblasts and calcifying VSMCs (Fig. 2D). Cell-associated elastin was detected in control VSMCs, calcifying VSMCs and osteoblasts at all stages (Fig. 2E). Consistent with the mRNA expression, control VSMCs displayed higher elastin levels (≤50%) than osteoblasts and calcifying VSMCs at day 14; no differences were observed at day 7 (Fig. 2E).

### 3.4 Marked differences in the ultrastructure of VSMC calcification and the mineralised bone nodules formed by osteoblasts

**Fig. 3:**
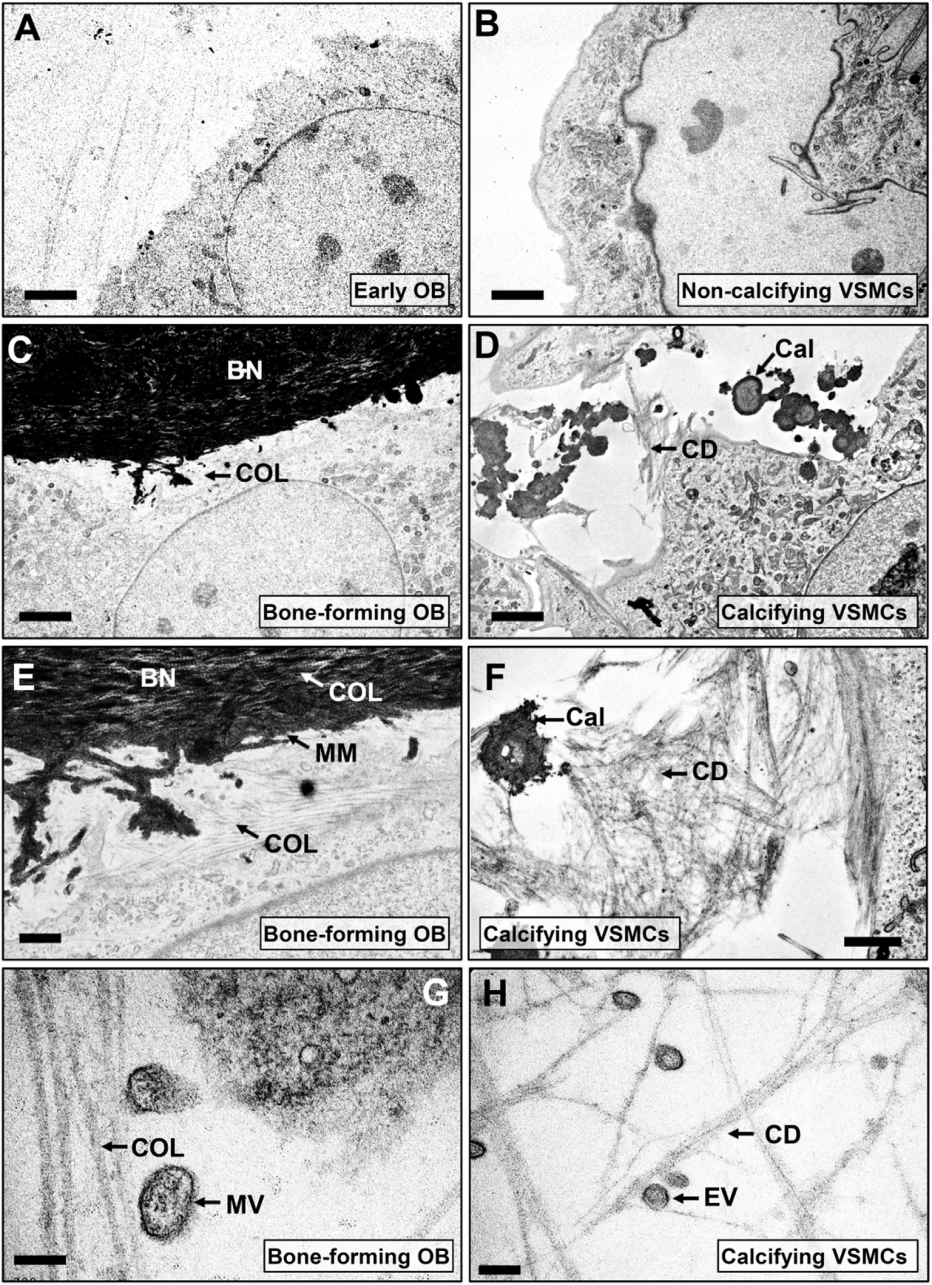
*Differences in the ultrastructure of VSMC calcification and the mineralised bone nodules formed by osteoblasts*. Cultures of **(A)** early osteoblasts and **(B)** non-calcifying VSMCs showed no evidence of any extracellular mineralisation. **(C)** In bone-forming osteoblasts there was widespread collagen [COL] deposition and clear mineralised matrix which formed part of a bone nodule [BN]. **(D)** Cultures of calcifying VSMCs contained amorphous regions of calcification [Cal] and extracellular fibril-like structures that appeared to be cellular debris [CD] but no deposited collagen fibres. **(E)** Higher power images showed that mineralised matrix [MM] was formed in and around the collagen fibres [COL] in bone-forming osteoblasts. **(F)** The extracellular calcification [Cal] present in VSMCs was less widespread, morphologically distinct from the osteoblast bone nodules and was sometimes associated with filamentous cellular debris [CD]. **(G)** In osteoblasts, matrix vesicles [MV] were seen associated with collagen fibres [COL]. **(H)** In calcifying VSMCs, extracellular vesicles [EV] were observed associated with filamentous cellular debris [CD]. Images shown are representative of the calcification and matrix deposition observed in three different cell preparations. Scale bars: A-B = 5  μm, C-D = 2 μm, E-F = 1  μm, G-H = 200 nm.

TEM imaging of early osteoblasts showed no evidence of bone formation (Fig. 3A). Clear extracellular deposition of collagen fibres and widespread mineralised matrix (Fig. 3C and E) were evident in mature, bone-forming osteoblasts. The banding pattern typically associated with collagen fibres was clearly visible within the mineralised bone nodule (Fig. 3E). No calcification was observed in VSMCs grown under basal conditions (Fig. 3B). In cultures of calcifying VSMCs, there were no collagen fibres, however, deposits of filamentous cellular debris were observed (Fig. 3D and F). VSMC calcification typically occurred as small discrete regions sometimes associated with cellular debris (Fig. 3F). Comparatively, osteoblast-mediated bone mineralisation was denser, larger and more widespread than the calcification in VSMC cultures (Fig. 3E and F). In bone-forming osteoblasts, matrix vesicles were observed in association with deposited collagen fibres, whilst cultures of calcifying VSMCs contained smaller extracellular vesicles that were often associated with the filamentous cellular debris (Fig. 3G and H).

### 3.5 TNAP activity is 100-fold higher in bone-forming osteoblasts than calcifying VSMCs

**Fig. 4:**
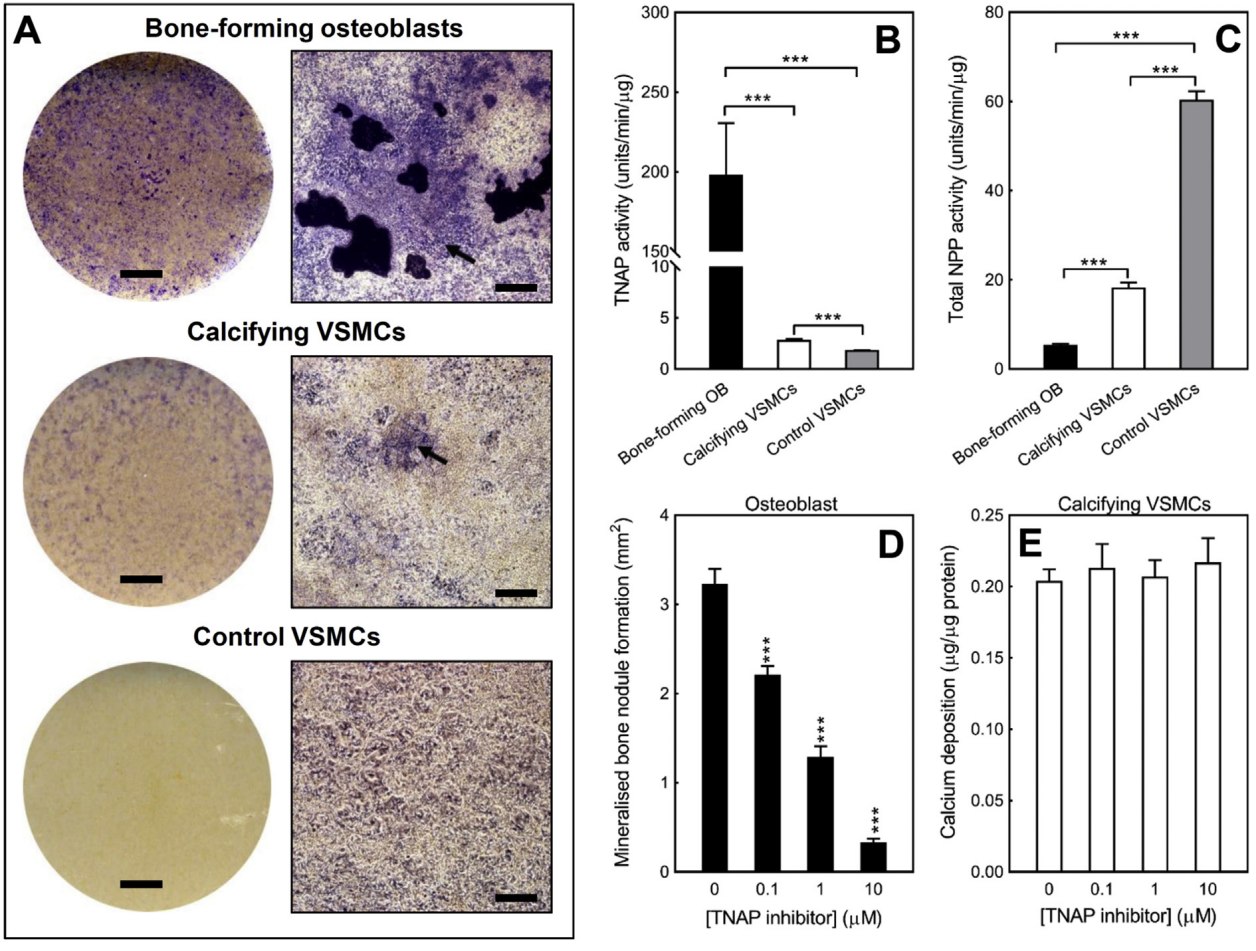
*TNAP activity is 100-fold higher in bone forming osteoblasts than calcifying VSMCs*. **(A)** Representative whole well scans and phase contrast microscopy images of osteoblasts and VSMCs stained to visualise TNAP expression. Bone-forming osteoblasts displayed the highest level of TNAP expression with only limited staining in calcifying VSMCs (highlighted by the arrow). No obvious TNAP staining was observed in control VSMCs. Scale bars: phase contrast microscopy images = 250 μm, tissue culture wells = 0.5 cm. **(B)** Rank order of TNAP activity was bone-forming osteoblasts >>> calcifying VSMCs > control VSMCs. TNAP activity was 50% higher in calcifying VSMCs compared to control VSMCs. **(C)** Rank order of total NPP activity was control VSMCs > calcifying VSMCs > bone-forming osteoblasts. **(D)** Culture with a TNAP inhibitor (≥0.1  μM) reduced bone formation by up to 90%. **(E)** Inhibition of TNAP had no effect on VSMC calcification. Values are mean ± SEM (*n* = 6 replicate wells), *** = p < 0.001.

Bone-forming osteoblasts showed strong expression of TNAP, particularly in the cells surrounding the bone nodules (Fig. 4A). In contrast, calcifying VSMCs displayed limited, diffuse staining for TNAP (Fig. 4A). No obvious TNAP staining was evident in cultures of control VSMCs (Fig. 4A). The rank order for TNAP activity was bone-forming osteoblasts >>> calcifying VSMCs > control VSMCs (Fig. 4B); VSMCs displayed a very low level of enzyme activity relative to osteoblasts (at least 100-fold lower). Basal TNAP activity was 50% higher in calcifying VSMCs (2.85 ± 0.1 units/min/μg) compared to control VSMCs (≤1.84 ± 0.1 units/min/μg) (Fig. 4B).

The TNAP inhibitor (CAS496014-13-2) concentration-dependently (≥0.1  μM) reduced bone formation by up to 90% (Fig. 4D). In contrast, culture of VSMCs with the same TNAP inhibitor had no effect on the level of calcification (Fig. 4E).

### 3.6 VSMCs display higher total NPP activity than osteoblasts

Rank order of total NPP activity was control VSMCs > calcifying VSMCs > bone-forming osteoblasts (Fig. 4C). NPP activity in calcifying VSMCs was 3-fold lower than control VSMCs. Osteoblast total NPP activity was 3.4-fold and 11-fold lower than calcifying and control VSMCs, respectively (Fig. 4C).

### 3.7 Cell number and viability are reduced in calcifying VSMCs but not in bone-forming osteoblasts

**Fig. 5:**
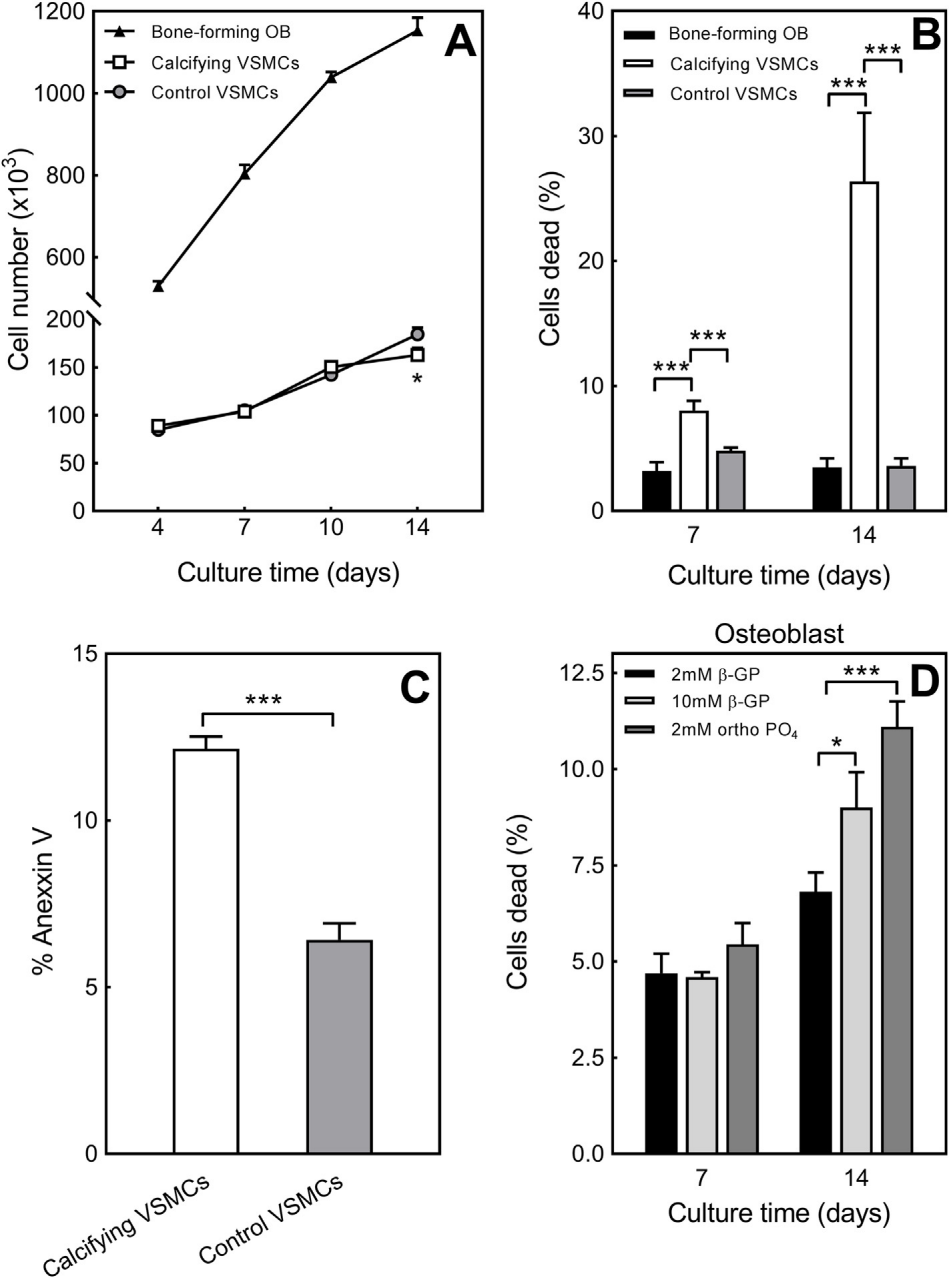
*Reduced cell viability and number in calcifying VSMCs*. **(A)** All cells showed an increase in number with increasing time in culture. Cell number was unchanged at day 4, 7 and 10 but was decreased 12% at day 14 in calcifying VSMCs compared to control VSMCs. **(B)** Increasing culture time was associated with a progressive reduction in calcifying VSMC cell viability; the percentage of dead cells was up to 7-fold higher in calcifying VSMCs compared to bone-forming osteoblasts and control VSMCs. **(C)** At day 7, the level of apoptosis was almost doubled in calcifying VSMCs compared to control cells. **(D)** Reduced viability in osteoblasts grown in medium associated with dystrophic mineral deposition. Values are mean ± SEM (*n* = 6 replicate wells), *** = p < 0.001, * = p < 0.05.

Both cell types progressively increased in number as the culture progressed; osteoblasts showed the largest increase in cell number over time (Fig. 5A). At day 4, 7 and 10 there were no differences in control and calcifying VSMC number; at day 14, cell number was 12% lower in calcifying VSMCs (Fig. 5A). Increasing culture time was also associated with a progressive reduction in viability (shown as percentage of dead cells) of calcifying VSMCs; osteoblast and control VSMC viability was unchanged (Fig. 5B). The percentage of dead cells was up to 7-fold higher in calcifying VSMCs than bone-forming osteoblasts and control VSMCs (Fig. 5B). The level of apoptosis was nearly doubled in calcifying VSMCs compared to control cells after 7 days in culture (Fig. 5C). For comparison, osteoblasts were also cultured in conditions associated with dystrophic mineral deposition (10 mM β-glycerophosphate or 2 mM sodium orthophosphate); these cells displayed reduced viability compared to osteoblasts grown in optimal conditions for regulated bone formation (+2 mM β-glycerophosphate) (Fig. 5D).

### 3.8 Comparison of marker gene expression between VSMCs and bone-forming osteoblasts

**Fig. 6:**
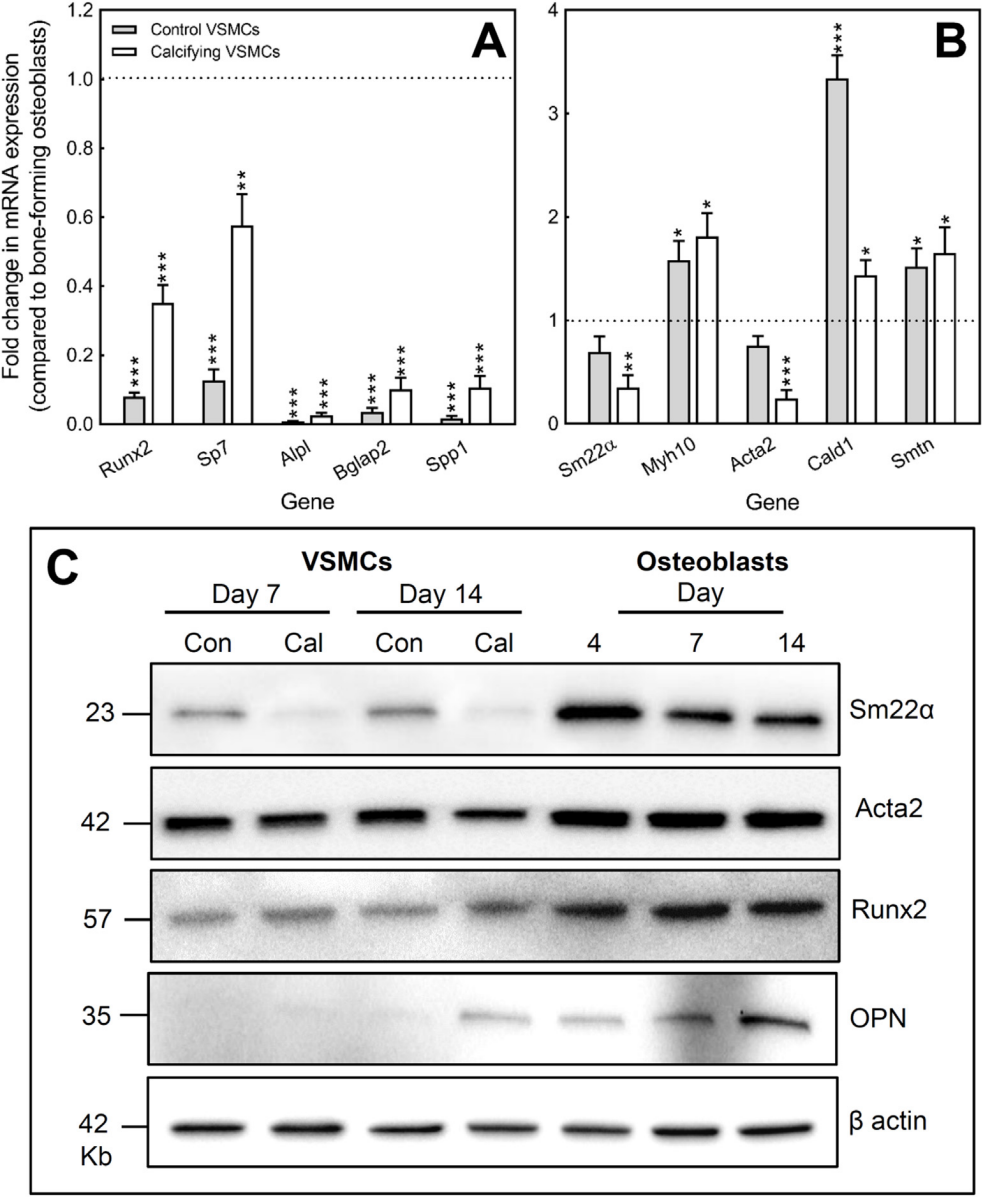
Comparison of marker gene expression in osteoblasts and VSMCs. **(A)** Expression of *Runx2, Sp7, Alpl*, *Bglap2* and *Spp1* was increased in calcifying VSMCs compared to control VSMCs but, in all cases, mRNA expression levels were lower in VSMCs than osteoblasts. **(B)** Osteoblasts express mRNA for all the VSMC marker genes studied. Only *Cald1, Myh10* and *Smtn* were expressed at a higher level in VSMCs than osteoblasts. mRNA expression is presented as the fold change in VSMC expression relative to bone-forming osteoblasts. Values are mean ± SEM (*n* = 4 RNA sets), *** = p < 0.001, ** = p < 0.01 * = p < 0.05. **(C)** Protein expression of Sm22α was lower in calcifying VSMCs compared to control VSMCs at both day 7 and 14; calcification had a limited effect on Acta2 levels. Osteoblasts also displayed strong expression of these proteins with Sm22α expression being higher in osteoblasts than VSMCs. Runx2 was detected in osteoblasts, control and calcifying VSMCs with the highest levels being seen in osteoblasts. OPN protein was expressed at all stages of osteoblast differentiation but protein levels were highest in mature, mineralising osteoblasts. OPN protein was only evident in calcifying VSMCs at day 14. Blots shown are representative of experiments performed 3 times with different protein sets.

mRNA expression of typical osteoblast (*Runx2*, *Sp7, Alpl, Spp1, Bglap2*) and VSMC (*Sm22α, Myh10, Acta2, Cald1, Smtn*) markers was investigated in control VSMCs, calcifying VSMCs and bone-forming osteoblasts after 14 days of culture. Data are presented as the fold change in mRNA expression relative to that seen in bone-forming osteoblasts (Fig. 6A and B).

Previous work has shown that VSMC calcification is associated with an increase in expression of osteoblast markers [10,13]. Consistent with this, we found that expression of *Runx2, Sp7, Alpl*, *Bglap2* and *Spp1* was increased up to 6-fold in calcifying VSMCs compared to control VSMCs (Fig. 6A). However, in all cases mRNA levels were significantly lower in VSMCs than in mature, bone-forming osteoblasts. Expression of *Runx2* and *Sp7* (early osteoblast markers) was up to 12-fold lower in control VSMCs and 3-fold lower in calcifying VSMCs compared to osteoblasts. Control VSMCs showed very limited expression of mature osteoblast marker genes (*Alpl, Bglap2, Spp1)* with mRNA levels up to 120-fold lower than that seen in bone-forming osteoblasts. Expression levels of these genes increased when VSMCs began to calcify but remained up to 40-fold lower than in osteoblasts (Fig. 6A).

Bone-forming osteoblasts were found to express mRNA for all the VSMC marker genes studied (Fig. 6B). Of these, only *Cald1, Myh10* and *Smtn* were expressed at a higher level in VSMCs than osteoblasts. Expression of *Sm22α* and *Acta2* was at a similar level in control VSMCs but up to 4-fold lower in calcifying VSMCs (compared to osteoblasts) (Fig. 6B).

### 3.9 Comparison of protein expression in osteoblasts and VSMCs

Changes in the protein expression of osteoblast (Runx2, OPN) and VSMC genes (Sm22α, Acta2) were investigated in osteoblasts and VSMCs. After 7 and 14 days of culture, levels of Sm22α were lower in calcifying VSMCs compared to control VSMCs (Fig. 6C). Calcification had less of an effect on Acta2 protein expression with only a small decrease at day 14. Osteoblasts also displayed strong expression of these proteins with Sm22α levels, in particular, being higher in osteoblasts than VSMCs. Furthermore, cellular differentiation appeared to influence osteoblast Sm22α expression since the highest levels were seen in proliferating cells (day 4) (Fig. 6C).

The transcription factor, Runx2, is considered an early osteoblast marker whilst OPN is a marker of more mature, bone-forming cells. Runx2 protein expression was detected in osteoblasts, control and calcifying VSMCS but, in keeping with the mRNA data, the levels were higher in osteoblasts (at all stages of differentiation). Interestingly, Runx2 expression was fairly similar in calcifying and control cells (Fig. 6C). OPN expression increased with osteoblast differentiation with the highest levels in mature, bone-forming cells. At day 7, control and calcifying VSMCs showed no OPN protein expression; however, low OPN expression was evident in calcifying VSMCs at day 14 (Fig. 6C).

### 3.10 Release of the mineralisation inhibitor, ATP, by osteoblasts and VSMCs

**Fig. 7:**
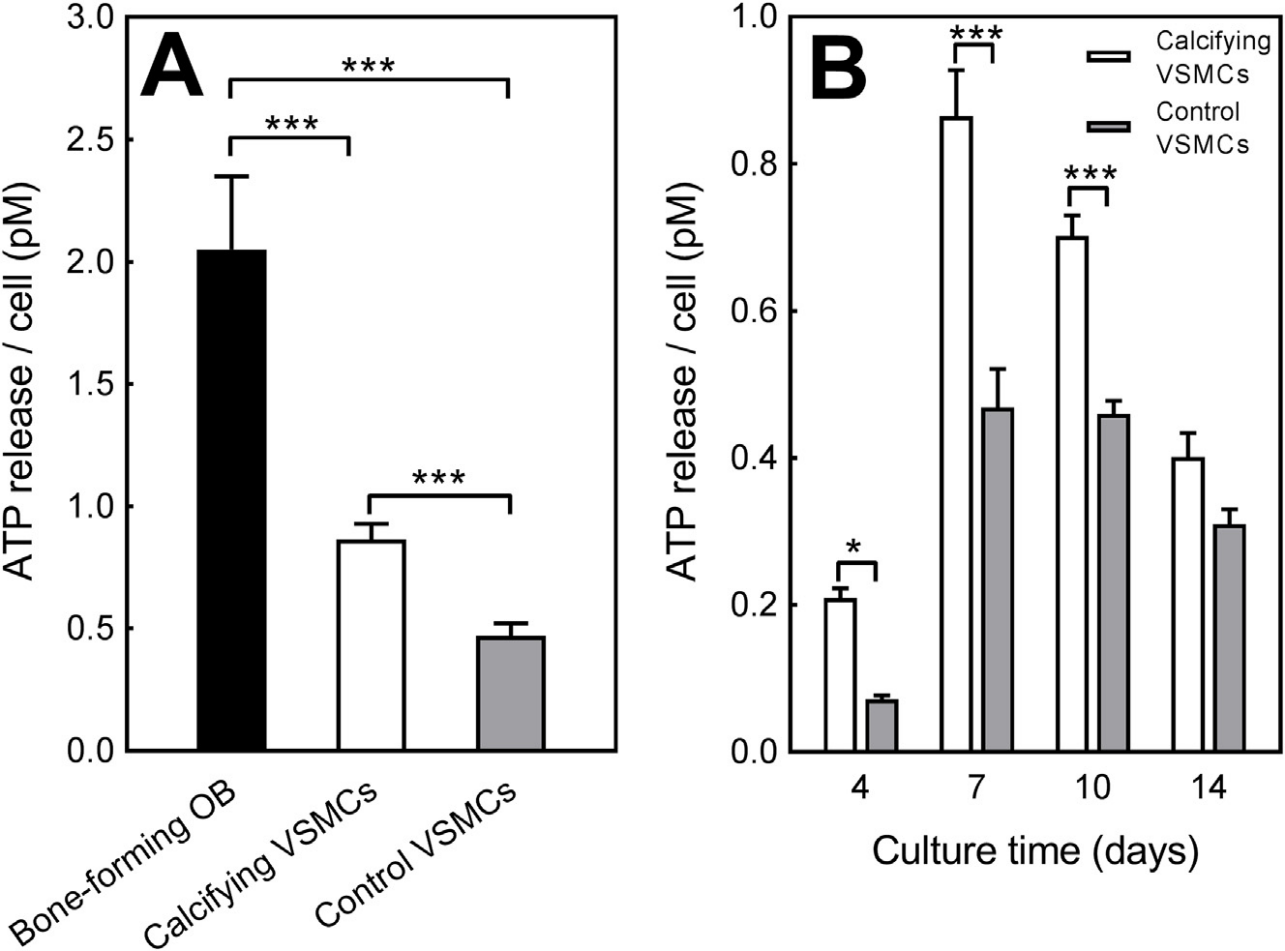
*Release of the mineralisation inhibitor, ATP, by osteoblasts and VSMCs*. **(A)** Rank order of ATP release per cell was bone-forming osteoblasts > calcifying VSMCs > control VSMCs. Osteoblasts released 2.4-fold and 4.3-fold more ATP than calcifying and control VSMCs, respectively. **(B)** Basal ATP release was up to 85% higher in calcifying VSMCs compared to control VSMCs. Values are mean ± SEM (*n* = 12 replicate wells), *** = p < 0.001, * = p < 0.05.

ATP is an extracellular signalling molecule and a substrate for a range of ecto-nucleotidases including NPP1. Side-by-side comparison after 14 days in culture showed that rank order of ATP release per cell was bone-forming osteoblast > calcifying VSMCs > control VSMCs; osteoblasts released 2.4-fold and 4.3-fold more ATP than calcifying and control VSMCs, respectively (Fig. 7A). Throughout the culture period, the extracellular ATP levels were up to 85% higher in calcifying VSMCs compared to control VSMCs (Fig. 7B).

### 3.11 Identification of a ‘universal’ mineralisation medium for osteoblastic bone formation and VSMC calcification

**Fig. 8:**
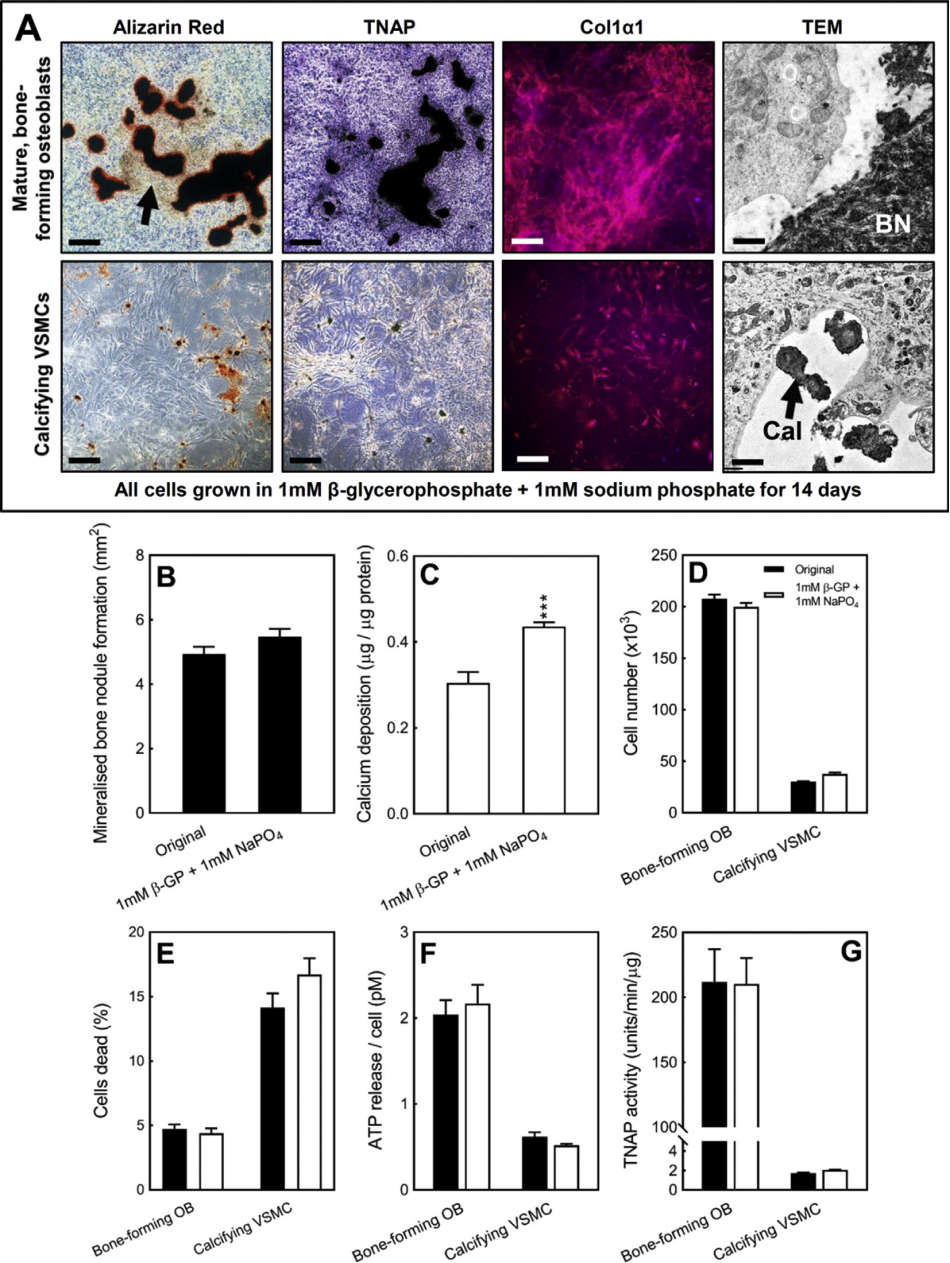
*The same functional differences in bone formation and VSMC calcification are observed when cells are grown in identical tissue culture conditions.***(A)** Osteoblasts cultured in 1 mM β-glycerophosphate + 1 mM sodium phosphate form large mineralised bone nodules and display widespread collagen deposition and TNAP staining. VSMCs grown in the same conditions form small discrete regions of calcification that are not associated with TNAP staining. Col1α1 immunostaining in VSMCs is cell associated (Scale bars: phase contrast = 250 μm, immunofluorescence = 50 μm, TEM = 1 μm). The level of **(B)** bone formation is similar in cells cultured under the original versus new conditions. **(C)** The level of VSMC calcification was increased by 40% in the new conditions. Comparison of **(D)** cell number, **(E)** cell viability, **(F)** ATP release and **(G)** TNAP activity shows that the functional differences between osteoblasts and VSMCs are the same in cells cultured in the original and new conditions. Original conditions: 2 mM β-glycerophosphate (osteoblasts) or 2 mM sodium orthophosphate (VSMCs). New conditions: 1 mM β-glycerophosphate + 1 mM sodium phosphate. Values are mean ± SEM (*n* = 6–12 replicate wells), *** = p < 0.001, ** = p < 0.01.

One potential limitation of the results described above is that they use slightly different tissue culture conditions to induce bone mineralisation/VSMC calcification (i.e. the form of phosphate used). To verify that the effects observed here were due to fundamental differences in the cells rather than the form of phosphate added, we identified conditions that could induce regulated bone formation and VSMC calcification. Culture of cells in medium supplemented with 1 mM β-glycerophosphate and 1 mM sodium diphosphate (1 + 1 conditions) resulted in selective mineralisation of the collagenous matrix in osteoblasts and the formation of small regions of calcification in VSMCs (Fig. 8A). For both cell types, the calcification formed displayed a similar morphology to that seen in osteoblasts and VSMCs cultured in the original conditions (Fig. 1A and F). The level of TNAP and Col1α1 staining and the appearance of the calcification under TEM also strongly resembled that observed with the original conditions (Fig. 8A).

The different cell culture conditions had no effect on the overall level of mineralised bone nodule formation (Fig. 8B), whilst the level of VSMC calcification was increased by 40% in the 1 + 1 conditions (Fig. 8C). Key parameters such as cell number (Fig. 8D), cell viability (Fig. 8E), ATP release (Fig. 8F) and TNAP activity (Fig. 8G) were unchanged. Furthermore, in all cases the differences between mineralising osteoblasts and calcifying VSMC were still observed.

## 4 Discussion

The development of AMC is widely accepted to be associated with a phenotypic transdifferentiation of VSMCs which results in them taking on characteristics usually associated with bone-forming osteoblasts [2,8,10,12]. The culture of VSMCs in a calcifying (high phosphate) environment is widely used to study the pathogenesis of AMC *in vitro.* This investigation directly compared this model of AMC with the established and extensively used osteoblast bone formation assay [[26], [27], [28]]. It also sought to develop a ‘universal’ calcification medium which allowed the direct comparison of both processes. We found that, compared to control VSMCs, calcifying VSMCs exhibit some limited osteoblast-like characteristics. However, they differ to mature osteoblasts in a number of key ways including: 1) their failure to form collagen-containing bone; 2) their lack of reliance on TNAP to promote mineral deposition; and, 3) the negative effect of calcification on their survival. Our study shows that development of VSMC calcification *in vitro* is a process distinct from the bone nodule formation performed by cultured osteoblasts. Furthermore, calcifying VSMCs appear to have a transitional phenotype that lies somewhere between that of a normal VSMC and a mature, bone-forming osteoblast.

VSMC calcification and mineralised bone nodule formation are the commonly used experimental end points for studying AMC and osteogenesis, respectively, *in vitro*. This investigation clearly shows that, when cultured with physiologically relevant levels of phosphate (i.e. ≤3 mM), these processes differ significantly. The data presented in Fig. 1, Fig. 2, Fig. 3, and also in many other studies [26,28,32], show that osteoblasts secrete and deposit significant amounts of collagenous matrix which is then mineralised to form large “trabecular-shaped” bone nodules *in vitro*. In contrast, culture of VSMCs in phosphate-containing medium led to the formation of numerous small regions of calcification that were not obviously associated with extracellular matrix. High power imaging using TEM revealed that this calcification was frequently located on and around filamentous cellular debris rather than collagen fibres. Further analysis of collagen production showed that both cell types expressed *Col1α1* mRNA and protein. In the case of VSMCs, and consistent with earlier work [33], Col1α1 expression was primarily localised in or on the surface of cells. Whilst clearly expressed by VSMCs, no soluble collagen was detected in the culture medium and no deposited collagen fibres were evident in the TEM images. This indicates that VSMCs are not exporting and depositing collagen into the extracellular environment in the same manner as osteoblasts. However, this lack of reliance on collagen for *in vitro* VSMC calcification is in contrast to a recent study which implicated the collagen cross-linking factors, LOX and PLOD, in the development of AMC [34]. Cell associated elastin was detected in both VSMCs and osteoblasts with the highest level seen in control, non-calcifying VSMCs. The observed decrease in elastin production in calcifying VSMCs compared to control cells is consistent with earlier studies which have associated AMC with a loss of elastin [35,36]. Taken together, these data show that the extracellular matrix produced by cultured VSMCs *in vitro* differs considerably in composition, abundance and structure to that which is secreted by bone-forming osteoblasts.

Earlier work has linked the initiation of AMC with increased VSMC apoptosis [15,37,38]. Although mechanistically not fully defined, it is thought that a calcifying environment can induce apoptosis and the VSMC-derived apoptotic bodies can then act as nucleation sites for hydroxyapatite crystal formation [15]. Consistent with these observations, we found that VSMC calcification was associated with higher levels of cell death and apoptosis compared to control VSMCs. The increase in calcifying VSMC apoptosis was measured after 7 days in culture, at this point the level of calcification is still fairly low and any changes in gene expression are less pronounced. This further supports the notion that increased apoptosis is one of the early events in the development of AMC. Earlier studies have also shown that inhibiting apoptosis can lead to a reduction in AMC [38,39]. In agreement, we have previously demonstrated that extracellular ATP and UTP can inhibit VSMC calcification by up to 80%, a protective effect that is mediated via the prevention of calcification-induced apoptosis [40]. However, since extracellular nucleotides do not fully block calcification, these findings also suggest that increased apoptosis is not the only factor driving the development of VSMC calcification in this experimental model.

In contrast, physiological bone formation is not associated with significant osteoblast cell death. Consistent with this, the viability of actively mineralising osteoblasts remained unchanged when cells were grown in optimal conditions for bone formation. Bone mineralisation is instead initiated within matrix vesicles where the coordinated actions of enzymes and proteins allow regulated hydroxyapatite formation without cell death [41,42]. Matrix vesicles associated with collagen fibres were evident in our bone-forming osteoblast cultures. AMC has also been linked with the release of mineralisation-competent extracellular vesicles [14]. Consistent with these findings, we observed extracellular vesicles associated with filamentous cellular debris. Together, these data suggest that the processes which drive the initiation of calcification in AMC are much more heterogeneous than those in bone mineralisation. Thus, the dissimilar appearance of VSMC calcification and mineralised bone nodules *in vitro* could reflect differences in both the extracellular matrix and the events which initiate calcification.

Bone mineralisation is a tightly regulated process that involves the coordinated activity of TNAP and NPP1 to control the phosphate/pyrophosphate ratio [20,21]. AMC has been associated with an increase in VSMC TNAP expression and activity [11,23,43]. Our data show that a high phosphate environment increases VSMC TNAP expression and activity to some extent. However, calcifying VSMCs still displayed levels of TNAP activity that were up to 100-fold lower than those observed in bone-forming osteoblasts. Furthermore, β-glycerophosphate (2–10 mM), which is hydrolysed by TNAP to produce phosphate, did not induce VSMC calcification within 14 days (unlike phosphate). Culture with a TNAP inhibitor also had no effect on the level of calcification. This observation is in agreement with earlier studies which showed no effect of TNAP inhibition on calcification [44,45] but is in contrast to other investigations which reported that blocking TNAP activity can reduce calcification [46,47]. The reasons for this discrepancy between investigations is unclear but may be related to the experimental approaches used. In particular, many studies use the non-selective compound Levamisole to reduce TNAP activity, here we used the more potent and selective TNAP inhibitor (CAS496014-13-2) [48]. The effectiveness of this compound is highlighted by the clear, dose-dependent inhibition of mineralised bone nodule formation in osteoblast cultures (Fig. 4E). Taken together our findings suggest that, unlike in osteoblasts, TNAP does not play a significant role in mediating VSMC calcification *in vitro*. Interestingly, we have also shown that extracellular ATP, at a concentration that inhibits VSMC calcification by up to 90%, actually causes a counter-intuitive increase in TNAP activity [40]. Therefore it is clear, that the exact role of TNAP in driving the development of VSMC calcification *in vitro* requires further clarification.

Under normal physiological conditions, VSMCs can prevent tissue calcification by producing local inhibitors such as pyrophosphate [2]. Previous studies have shown that development of AMC is associated with a decrease in NPP1 activity [18,24,25]. Consistent with this, we found that control VSMCs displayed the highest level of total NPP activity and calcification was associated with a loss of enzyme activity.

It has been reported that calcifying VSMCs undergo a phenotypic transdifferentiation to become more osteoblast-like [10,12]. These changes have been associated with increased expression of typical osteoblast genes (e.g. *Runx2*, *Alpl* (TNAP), *Bgalp2* (OCN), *Spp1* (OPN)) and decreased expression of VSMC genes (e.g. *Sm22α, Acta2*) [10,12]. This study directly compared the relative levels of gene expression between mature, bone-forming osteoblasts, control VSMCs and calcifying VSMCs for the first time. An increase in osteogenic gene expression in calcifying VSMCs was observed in this study; however, the relative levels of mRNA expression remained significantly lower in calcifying VSMCs than osteoblasts. Markers of the mature osteoblast phenotype such as *Alpl, Bgalp2* and *Spp1* showed particularly limited expression. The low mRNA levels of these genes suggests that, even when there is widespread calcification, calcifying VSMCs are only at the early stages of differentiating towards an osteoblast-like lineage.

VSMCs show a high degree of plasticity and therefore many genes are used as markers of the cell phenotype including *Sm22α, Acta2, Cald1, Myh10* and *Smtn* [49,50]. In broad agreement to earlier work, we observed decreased levels of *Sm22α*, *Acta2* and *Cald1* mRNA in calcifying VSMCs. Somewhat surprisingly, we found that bone-forming osteoblasts also expressed mRNA for all of the VSMC genes studied. In general, the relative level of mRNA expression was higher in VSMCs than osteoblasts; however, in the case of *Sm22α* and *Acta2*, bone-forming osteoblasts expressed more mRNA than calcifying VSMCs. Additionally, strong Sm22α and Acta2 protein expression was observed in osteoblasts at all stages of differentiation, and in the case of Sm22α levels were higher in osteoblasts than VSMCs. These findings are consistent with previous studies which reported expression of VSMC markers (e.g. Acta2, calponin, Myh10) by osteoblasts and mesenchymal stem cells [[51], [52], [53], [54], [55], [56]]. Thus, despite being widely used in publications, these proteins may not represent the most appropriate markers for studying the loss of/change in VSMC phenotype in a calcifying environment.

Controlled ATP release has been demonstrated from many cell types including VSMCs and osteoblasts [31,57]. This study found that extracellular ATP levels were higher in cultures of calcifying VSMCs than control VSMCs. ATP can act via P2 receptors to inhibit bone mineralisation [29,58] and AMC [40,59]. Furthermore, ATP can also be broken down by NPPs to produce pyrophosphate. Thus, the increase in ATP release from calcifying VSMCs could reflect a mechanism by which the cells are attempting to prevent further calcification by releasing local inhibitors. However, since calcifying VSMCs also display reduced cell viability the higher extracellular ATP levels could also reflect the release of intracellular ATP from dying cells.

Many of the experimental protocols employed to model AMC *in vitro* do so by increasing the extracellular phosphate levels in the culture medium. The additional phosphate added varies from 2mM to 10mM and is usually in the form of sodium phosphate [40,60,61] or, occasionally, β-glycerophosphate [10,39]. Even in severe hyperphosphatemia, serum phosphate levels of over 3 mM are rare *in vivo;* thus, the experimental study of higher phosphate concentrations may have little pathophysiological relevance. Furthermore, a recent investigation by Hortells et al. [62] reported that higher concentrations of phosphate (≥3 mM) will saturate the tissue culture medium leading to non-specific, non-cell mediated calcium phosphate precipitation. The same also applies to osteoblasts since culture with ≥5 mM β-glycerophosphate leads to reduced cell viability and widespread non-specific mineralisation that is not true bone formation (Figs. 1 and 5) [26,28]. Thus, to avoid confounding results caused by dystrophic mineral deposition, this study used low, physiologically relevant phosphate concentrations (2 mM) to compare AMC and bone formation. Since β-glycerophosphate failed to induce VSMC calcification within 14 days, sodium orthophosphate was used in the VSMC experiments. Sodium orthophosphate was not suitable for osteoblast cultures because, even at 2 mM, it resulted in non-specific mineral deposition and increased cell death. Both of these events are not representative of the *in vivo* physiological situation and therefore not appropriate to use. The comparison of cells grown in different conditions represents a potential limitation of the findings presented as it is possible that the differing forms of phosphate contributed to some of the functional differences observed. Therefore, we also sought to identify culture conditions which allow both regulated bone formation and VSMC calcification. A combination approach of 1 mM β-glycerophosphate + 1 mM sodium phosphate proved to be the most effective approach. Osteoblasts and VSMCs cultured using this medium supplementation behaved in the same way as cells grown in the original culture conditions. Taken together, this suggests that the functional differences observed between *in vitro* bone formation and VSMC calcification are most likely cell-mediated rather than due to the culture conditions.

In summary, this study showed that VSMCs cultured in a high phosphate environment undergo phenotypic changes to develop some limited osteoblast-like properties. However, calcifying mouse VSMCs differ significantly from mature, bone-forming mouse osteoblasts and instead appear to display a phenotype that is in between that of a normal cultured VSMC and a mature osteoblast. It should be emphasised that this comparison is for *in vitro* studies where calcification has been induced solely by increasing extracellular phosphate levels. However, given the widespread use of *in* vitro models in vascular calcification research it is important that the differences between osteoblasts and calcifying VSMCs are acknowledged in order to prevent inaccurate or over interpretation of research findings. Ultimately, understanding the differences between VSMCs and osteoblasts may help develop therapeutic strategies which prevent pathological AMC without impacting physiological skeletal mineralisation.

## Conflicts of interest

The authors have no conflict of interest.
